# Giant Sternal Chondrosarcoma in a 50-Year-Old Patient

**DOI:** 10.3390/healthcare10010158

**Published:** 2022-01-14

**Authors:** Cezar Pavelescu, Alexandru Bebliuc, Rareș Asmarandei, Maria Sabina Safta, Ondin Zaharia, Victor Sebastian Costache, Adrian Molnar, Daniela Gheorghiță, Cristian Voica, Horațiu Moldovan

**Affiliations:** 1 Bucharest Clinical Emergency Hospital, 014461 Bucharest, Romania; cezarpavelescu@hotmail.com (C.P.); bebliuc@gmail.com (A.B.); asmarandei.rares@yahoo.com (R.A.); mariasabinasafta@gmail.com (M.S.S.); cristianvoica@yahoo.com (C.V.); 2 Faculty of Medicine, Carol Davila University of Medicine and Pharmacy, 050474 Bucharest, Romania; ondin.zaharia@gmail.com; 3 Prof. Dr. Theodor Burghele Clinical Hospital, 061344 Bucharest, Romania; 4 Sf. Constantin Hospital, 500388 Brasov, Romania; victorscostache@gmail.com; 5 Faculty of Medicine, Titu Maiorescu University, 040441 Bucharest, Romania; 6 Faculty of Medicine, Iuliu Hatieganu University of Medicine and Pharmacy, 400000 Cluj-Napoca, Romania; adrianmolnar097@gmail.com; 7 Heart Institute, 400001 Cluj-Napoca, Romania; 8 Faculty of Materials Science and Engineering, Politehnica University of Bucharest, 060042 Bucharest, Romania

**Keywords:** sternal chondrosarcoma, chest wall reconstruction, primary malignant bone cancer

## Abstract

Chondrosarcomas represent approximately 20% of primary malignant bone cancers, being known as the most frequent neoplasia of the anterior thoracic wall. In our case, we present a case of a primary sternal chondrosarcoma in a 50-year-old female patient that has been polychemiotherapy and radiotherapy treated for breast cancer. Despite the initial treated malignancy of breast cancer in the personal pathologic history of the patient, it was discovered that the sternal tumor was not a metastatic disease from the breast neoplasm. After multiple investigations, the patient was successfully treated for the sternal chondrosarcoma after a radical sternal resection with a chest wall reconstruction completed with two titanium plates that were anchored on the ribs and with the placement of methyl methacrylate mesh.

## 1. Introduction

Primary malignant tumors of the thoracic wall are quite rare, representing approximately 1% of all neoplasms [1]. Chondrosarcoma represents the most frequent thoracic wall tumor, making up around 20% of the total cases, and from the medical literature we know that a sternal origin is four times rarer than a cartilaginous origin [2,3]. As the gold standard, a complete resection of the primary tumor is the best therapeutical option for sternal chondrosarcomas today [3,4].

## 2. Case Presentation

We present a rare clinical case of a 50-year-old female patient with a significantly large sternal chondrosarcoma and a vast pathological personal history until the moment of diagnosis.

It is known that the patient was operated on in 2010 for a left breast adenocarcinoma, for which she underwent both radiotherapy and chemotherapy with good prognosis. However, in 2016 the patient presented in the emergency room with cardiac tamponade. The first thought was that this status was related to the initial disease; however, postoperative (percutaneous drainage with biopsy) the metastatic pericarditis suspicion was declined.

The CT scan showed a sternal lesion at the level of the sternal manubrium, very suggestive of secondary lesions from the breast adenocarcinoma. The size of this sternal lesion grew in size, according to the repeated CT scans from 2017 and 2018. In 2019, the patient underwent a PET-CT scan that showed multiple secondary lesions at the level of the regional lymph nodes, muscle tissue and thoracic bones. Throughout these two years, the patient continues to undergo chemotherapy for the breast cancer.

**Figure 1:**
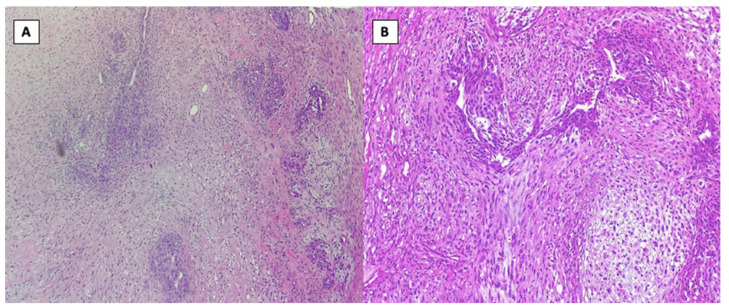
Histopathology images from the biopsy, H&E stain: (**A**)—20× and (**B**)—40× resolution.

In September 2020, due to a sternal tumor of large size, the patient developed a left lung pleurisy. During the management of this episode of pleurisy, another biopsy was taken from one of the punctures and, surprisingly, on the result of this pathology report the diagnosis of chondrosarcoma was established, as seen in Figure 1A,B.

**Figure 2:**
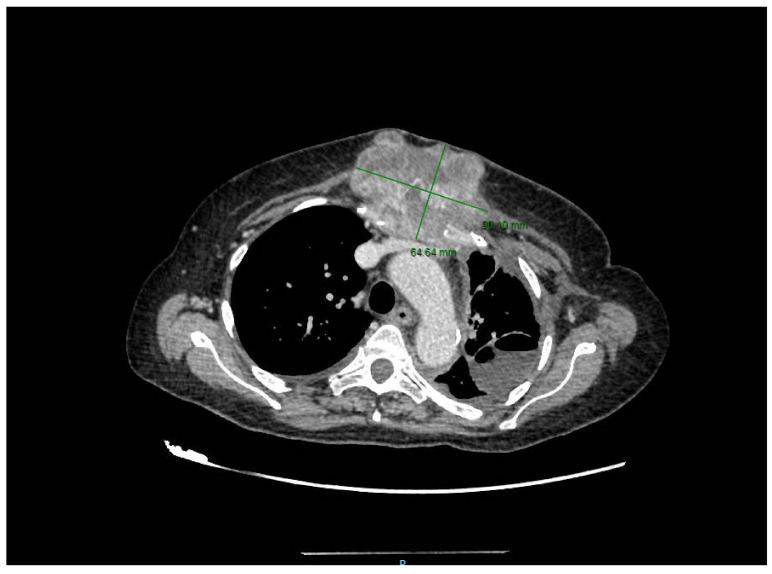
Initial CT aspect.

A month later, the patient returned with cardiac tamponade, and the emergency CT examination showed a sternocostal tumor of a large size (6/9 cm) and left pleurisy, as seen in Figure 2.

**Figure 3:**
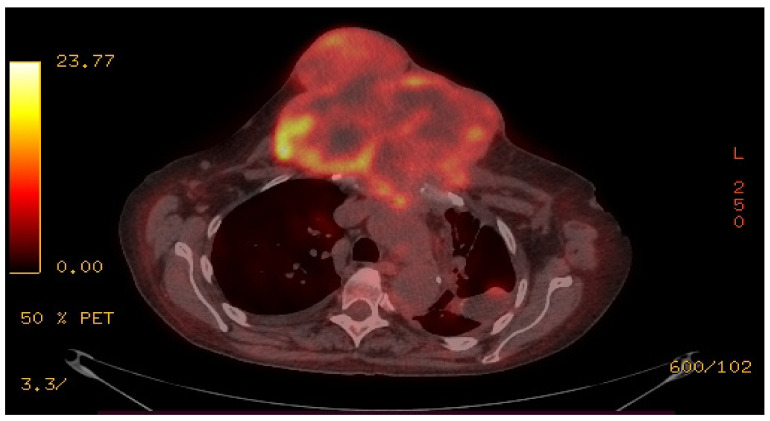
PET-CT scan.

Despite having respiratory discomfort, the patient refused surgical intervention and continued with the chemotherapy. Meanwhile, she underwent a PET-CT scan (Figure 3), that revealed an irregular-shaped mass, arising from the manubrium sterna. On the basis of the anatomic and metabolic findings, the PET-CT interpretation strongly indicated a well-differentiated malignant tumor.

Due to the cutaneous tumoral invasion, local bleeding occurred. The patient returned to our service and required emergency surgery. 

As the patient’s condition was deteriorating, a multidisciplinary team composed of thoracic, plastic and cardiovascular surgeons was formed.

The last preoperative CT scan shows the following: a large sternal tumoral process with an axial diameter of approximately 16 cm and vertical diameter of 15 cm that destroyed most of the sternal body. Furthermore, the tumor protrudes anteriorly in the subcutaneous tissue of the pre-sternum; and posteriorly it protrudes into the mediastinum, compressing on the ascending aorta, the left brachiocephalic trunk and the pericardium. The tumoral process infiltrates the left internal mammary vascular bundle. There is a 1.5 cm thick line of pericardial fluid and a bilateral pleurisy of approximately 1.5–2 cm (CT images provided in Figure 2). 

From the histopathology result, the diagnosis of chondrosarcoma was established, having CD 34, S100 and p63 markers positive (highly suggestive of chondrosarcomas). In Figure 1A,B, we can observe results with a 20× and 40× resolution from the HE slides obtained on the pathology report. 

After the full pathology report was received, the final diagnosis of Giant Sternal Chondrosarcoma was established. In Figure 1A,B, slides with H&E stain with a 20× and 40× magnification can be observed, these images being highly suggestive of chondrosarcoma. Furthermore, markers CD34, S100 and p63 turned out to be positive, another pathognomonic sign pointing towards the certain diagnosis of chondrosarcoma.

**Figure 4:**
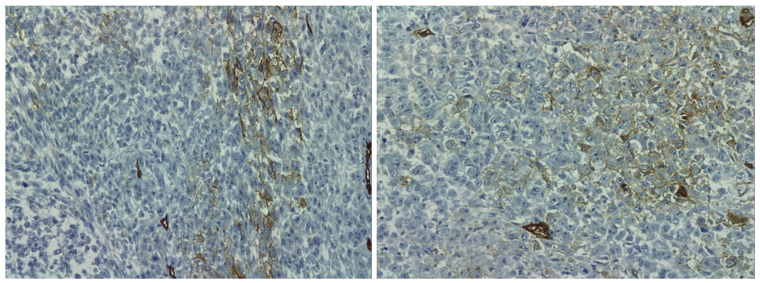
Immunohistochemistry images showing marker CD 34 positive for sternal chondrosarcoma.

**Figure 5:**
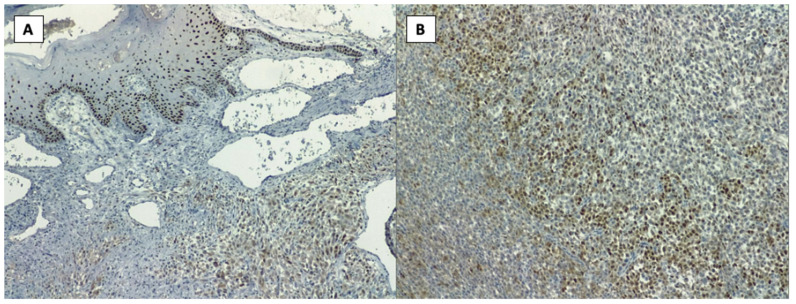
Immunohistochemistry images showing marker p63 positive for sternal chondrosarcoma: (**A**)—20× and (**B**)—40× resolution.

**Figure 6:**
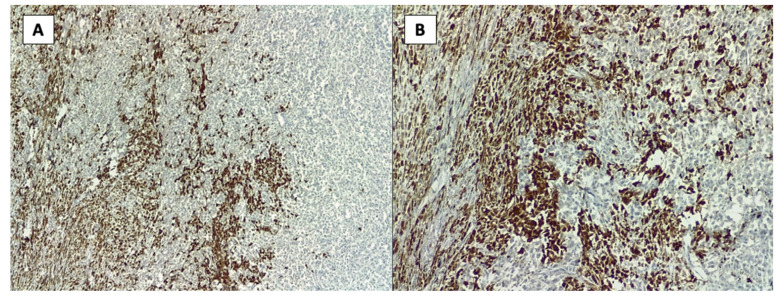
Immunohistochemistry images showing marker S100 positive for sternal chondrosarcoma: (**A**)—20× and (**B**)—40× resolution.

Below, we introduce the results from the immunohistochemistry analysis (Figure 4, Figure 5 and Figure 6).

The primary role of immunohistochemistry is for the definite diagnosis of the primary tumor of sternal chondrosarcoma. In our case, the laboratory used three markers: CD34, S100 and p63 that turned out to be positive and seen on the different coloring provided in the images in Figure 4, Figure 5 and Figure 6. CD34 is an endothelial marker sensitive in endothelial differentiation, which is positive in over 70% of sarcomas. S100 is a protein present in peripheral and central systems and always coexists with cytokines in sarcomas. While the p63 scored positive which outlines a high intensity staining with more than 50% tumor cells present.

**Figure 7:**
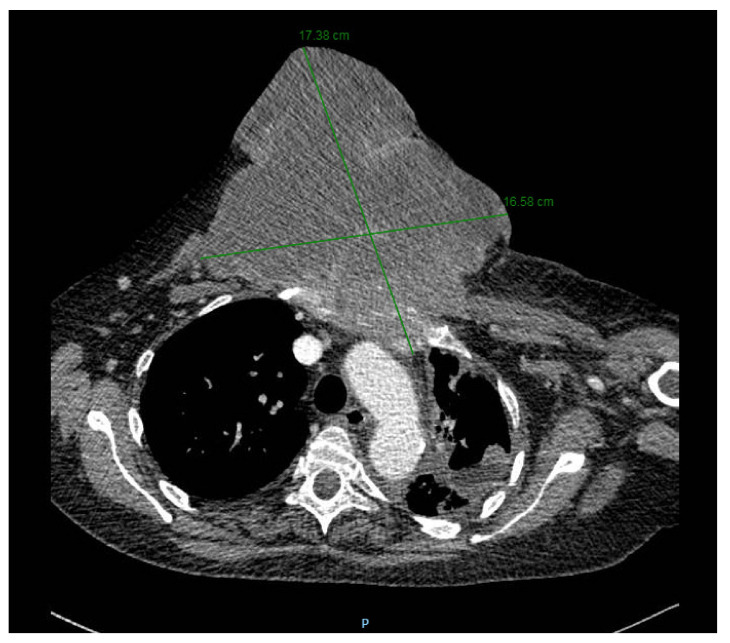
Preoperative CT scan (transversal view).

**Figure 8:**
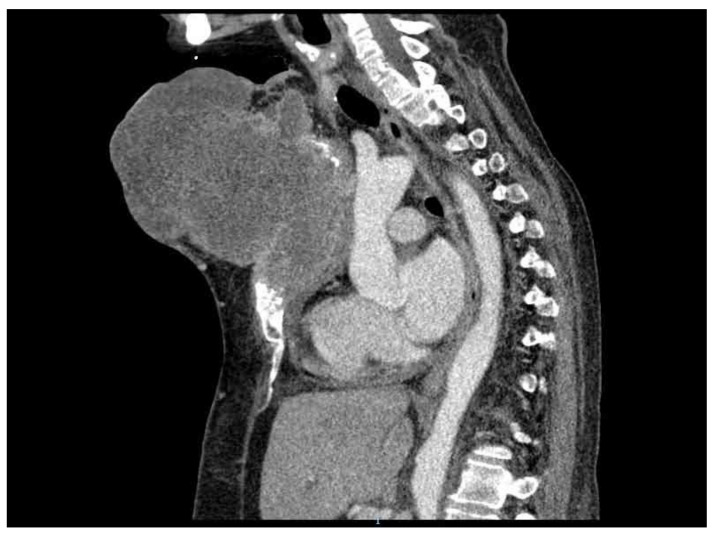
Preoperative CT scan (sagittal view).

**Figure 9:**
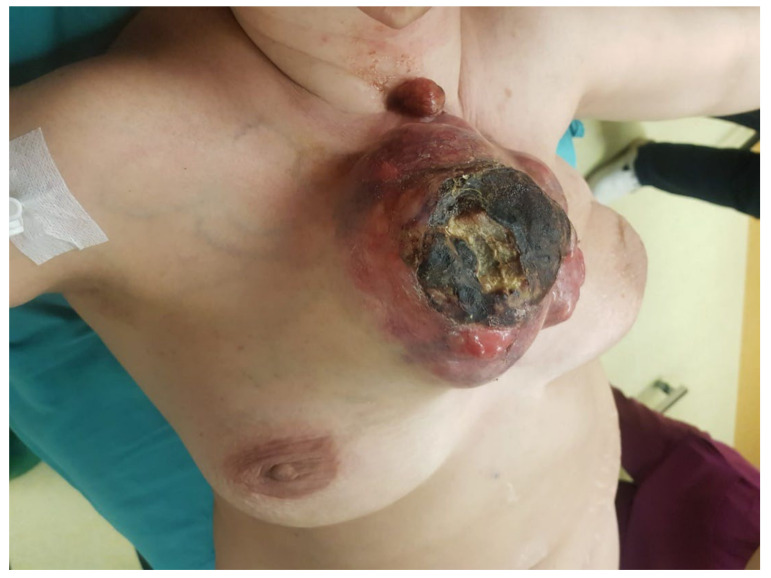
Preoperative aspect of the tumor.

Figure 7 presents the preoperative CT scan in transversal plane, while Figure 8 presents the preoperative CT scan in sagittal plane. The preoperative aspects of the tumor is presented in Figure 9.

A surgical approach using two teams of surgeons operating bilaterally was implemented, in order to shorten the surgery time as much as possible.

The operative team managed to take out the superior two-thirds of the sternum, the anterior margin of ribs 1, 2 and 3 and all the muscular tissue and skin layers invaded by the tumor. Luckily, the left brachiocephalic trunk was not invaded by the tumoral process, and no major bleeding occurred during the surgery.

In order to close the wound, we fixed the thoracic wall with two titanium plates, which were anchored on the ribs, with placement of methyl methacrylate mesh. The plastic surgeon repaired the parietal defect with the aid of the pectoral muscles, and the cutaneous defect by using a small skin graft and doing a breast lift. 

**Figure 10:**
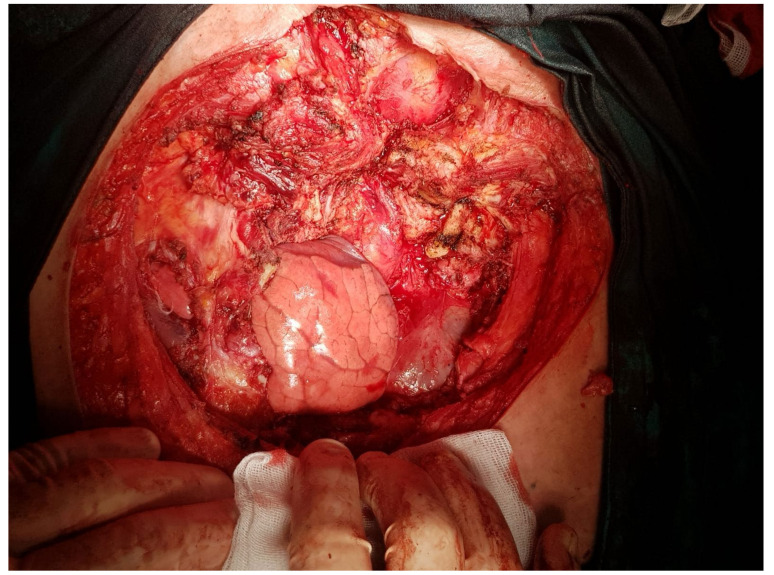
Intraoperative aspect, after tumor resection.

**Figure 11:**
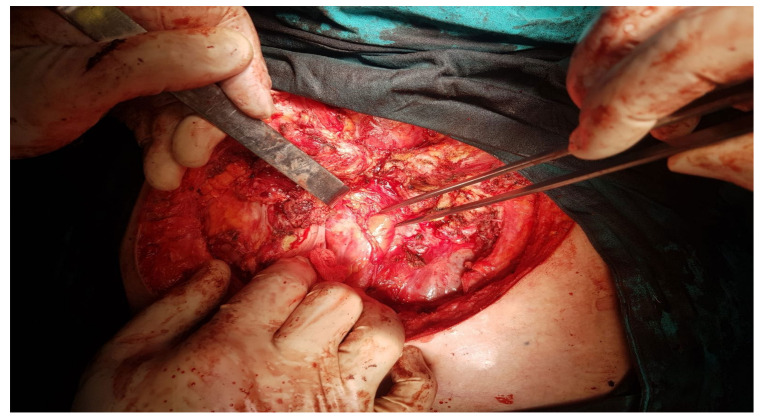
The isolated left brachiocephalic trunk, during surgery.

**Figure 12:**
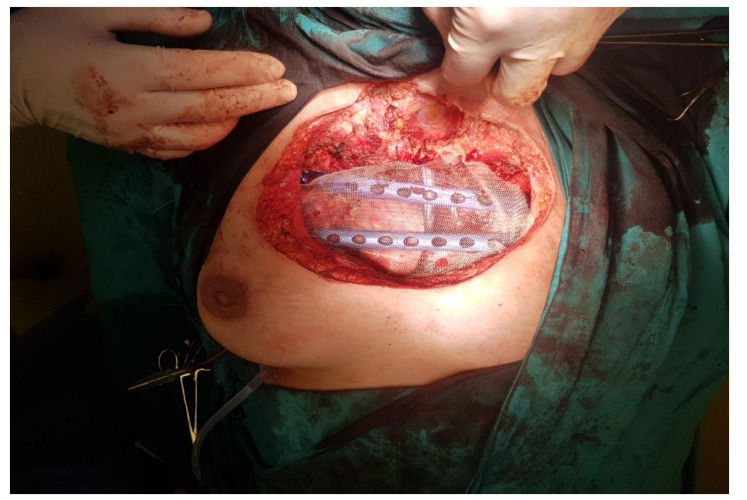
Thoracic wall reconstruction aspect.

**Figure 13:**
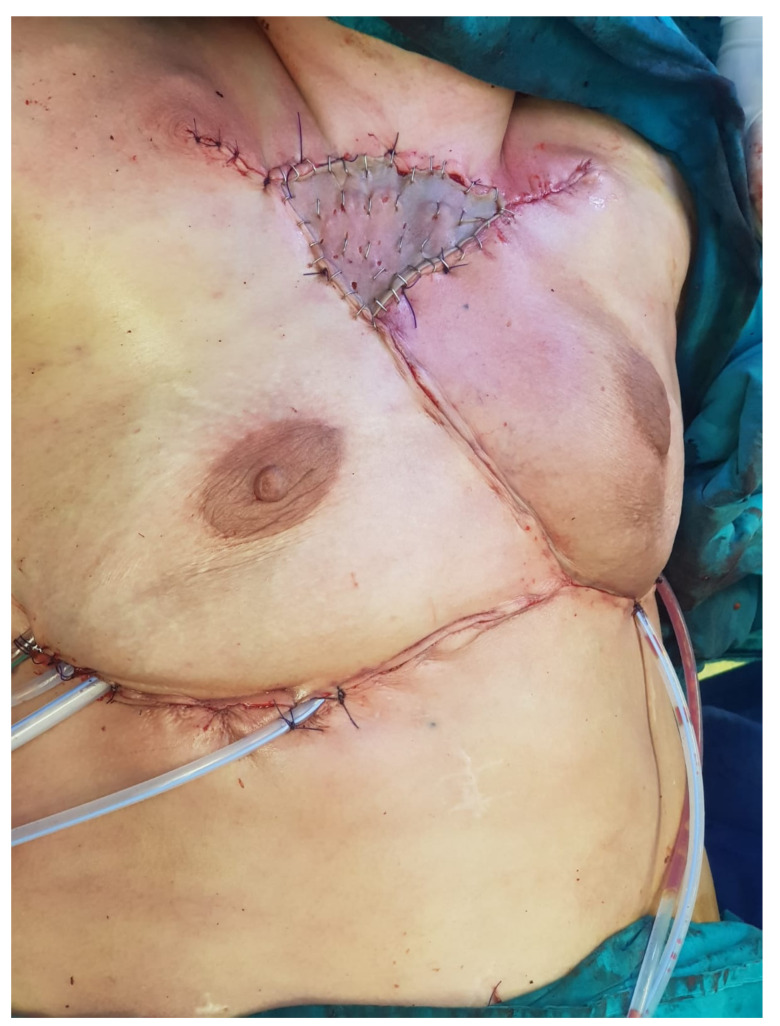
Final postoperative aspect.

The intraoperative aspect after tumor resection is presented in Figure 10. Figure 11 presents the intraoperative aspect of the isolated left brachiocephalic trunk. In addition, the aspect of the thoracic wall reconstruction is shown in Figure 12. Figure 13 presents the postoperative aspect.

Below, we present a few intraoperative images.

## 3. Discussion

Chondrosarcomas represent approximately 30% of primary malignant bone cancers, the most frequent being that of the anterior thoracic wall [4,5]. This type of tumor develops more often between the third and fourth decade of life [6,7]. What is concerning in this case, is the fact that the patient developed this condition after being treated for breast cancer, and it was not secondary to the first malignancy [8,9,10].

Among the primary chondrosarcomas, 85–90% are represented by conventional chondrosarcomas, with central, periosteal and peripheral subtypes; these have a low proliferation rate. The remaining 10–15% are represented by high-grade and differentiated chondrosarcomas, with a much worse prognosis than the other ones [5].

Chondrosarcomas represent a group of tumors resistant to both radiotherapy and chemotherapy, the reasons given being the low rate of mitosis and lack of tumor vascularization, which would aid in the transport of chemotherapeutic agents intratumorally. However, a number of therapies are being studied for this disease. Thus, variants of immunotherapy, epigenetic inhibitors (hypomethylating agents, HDAC) and targeted therapies (targeted therapies: angiogenesis inhibitors, CDK inhibitors, osteoclast inhibitors, tyrosine kinase inhibitors, and, quite promisingly, IDH-1 inhibitors) are being studied [11,12].

However, at this point in time, the main therapeutic option remains resection within oncological limits, especially for grade 1 chondrosarcomas, as in the case we have presented [13,14].

Thoracic wall chondrosarcomas typically grow at a slower rate and relapse first locally [11]. However, if not treated readily, late metastasis will occur as well. Thus, complete resection of the primary neoplasia is the main determinant of survival [12,13]. The purpose and aim of the surgical intervention must be a wide resection, in order to increase the quality of life of the patient [3,14]. A multidisciplinary team is considered to be of great importance in complex cases as it offers a balanced and complementary approach for optimal patient care [15]. 

In our clinical case, the biomaterials used were two titanium plates and a methyl methacrylate mesh. We know from the medical literature that such prosthetic mesh allows for long-lasting tolerability and the in-growth of regenerative tissues [16]. The methyl methacrylate mesh provides more core support and malleability for the chest wall defect. In order to provide stability and rigidity, two additional titanium plates were used [16]. 

Today, the materials used for thoracic wall reconstruction are being widely discussed [17,18]. Many approaches have been tried by surgeons worldwide [19]. At the end of the day, what is the most crucial aspect is that the thoracic wall retains some stability after the surgery [20,21]. We used titanium plates and the methyl methacrylate mesh for maximum stability on our patient, taking into account the number of interventions and procedures she underwent in recent years [22,23]. Regarding case particularity, we can state that we had a case of a primary sternal chondrosarcoma that was treated efficiently. This pathology was not secondary to the patient chemo-treated breast carcinoma, as initially expected.

## 4. Conclusions

In conclusion, by means of this article, we have presented a rare case of primary chondrosarcoma of the sternum, which was successfully treated by radical resection and reconstruction using titanium plates and methyl methacrylate mesh. The patient had a favorable postoperative evolution and was discharged 7 days later. The patient remains in contact with her doctors and should return for a follow-up every 6 months.

## Data Availability

The study did not report any data.
